# The Causes and Evolutionary Consequences of Mixed Singing in Two Hybridizing Songbird Species (*Luscinia* spp.)

**DOI:** 10.1371/journal.pone.0060172

**Published:** 2013-04-05

**Authors:** Jana Vokurková, Tereza Petrusková, Radka Reifová, Alexandra Kozman, Libor Mořkovský, Silke Kipper, Michael Weiss, Jiří Reif, Paweł T. Dolata, Adam Petrusek

**Affiliations:** 1 Department of Ecology, Faculty of Science, Charles University in Prague, Prague, Czech Republic; 2 Department of Zoology, Faculty of Science, Charles University in Prague, Czech Republic; 3 Institute of Biology, Animal Behaviour Group, Free University Berlin, Berlin, Germany; 4 Institute for Environmental Studies, Faculty of Science, Charles University in Prague, Prague, Czech Republic; 5 South Wielkopolska Group of the Polish Society for the Protection of Birds, Ostrów Wielkopolski, Poland; Utrecht University, Netherlands

## Abstract

Bird song plays an important role in the establishment and maintenance of prezygotic reproductive barriers. When two closely related species come into secondary contact, song convergence caused by acquisition of heterospecific songs into the birds’ repertoires is often observed. The proximate mechanisms responsible for such mixed singing, and its effect on the speciation process, are poorly understood. We used a combination of genetic and bioacoustic analyses to test whether mixed singing observed in the secondary contact zone of two passerine birds, the Thrush Nightingale (*Luscinia luscinia*) and the Common Nightingale (*L. megarhynchos*), is caused by introgressive hybridization. We analysed song recordings of both species from allopatric and sympatric populations together with genotype data from one mitochondrial and seven nuclear loci. Semi-automated comparisons of our recordings with an extensive catalogue of Common Nightingale song types confirmed that most of the analysed sympatric Thrush Nightingale males were ‘mixed singers’ that use heterospecific song types in their repertoires. None of these ‘mixed singers’ possessed any alleles introgressed from the Common Nightingale, suggesting that they were not backcross hybrids. We also analysed songs of five individuals with intermediate phenotype, which were identified as F_1_ hybrids between the Thrush Nightingale female and the Common Nightingale male by genetic analysis. Songs of three of these hybrids corresponded to the paternal species (Common Nightingale) but the remaining two sung a mixed song. Our results suggest that although hybridization might increase the tendency for learning songs from both parental species, interspecific cultural transmission is the major proximate mechanism explaining the occurrence of mixed singers among the sympatric Thrush Nightingales. We also provide evidence that mixed singing does not substantially increase the rate of interspecific hybridization and discuss the possible adaptive value of this phenomenon in nightingales.

## Introduction

Understanding the evolution of reproductive barriers preventing gene flow between incipient species is crucial for understanding the speciation process. Sexually selected traits in general, and those that are culturally transmitted in particular, are considered to play an important role in the origin of prezygotic reproductive isolation [1]. Bird song is one of these traits and its role in the establishment and maintenance of prezygotic barriers in birds seems to be crucial [2]–[6].

Depending on the type of interspecific interactions, sympatrically occurring species (including closely related species coming into secondary contact) can show divergence or convergence in vocalization patterns, as well as in other key characteristics of species recognition [7]. These may diverge as a result of natural selection, in order to avoid maladaptive hybridization [8] or to reduce interspecific competition [7], [9], [10]. On the other hand, species may also converge in some features; this is often observed in bird songs (e.g., [6], [11]). The mechanisms leading to song convergence may include (1) cross-species song learning [3], [12], [13], (2) ecological adaptation to the local environment [14]–[17], and (3) genetic introgression [18]–[20]. Such convergence can be followed by broad heterospecific pairing, resulting in increased interspecific hybridization and mixing of species gene pools [21]–[23].

Song in passerine birds is usually learned through an imprinting-like process, although a genetic component of song inheritance has also been described in some species [5], [24]. A predominantly cultural transmission of song may cause its rapid divergence in allopatry and thus accelerate the speciation process [25]–[27]. The same property of song can, however, oppose the speciation in sympatry if heterospecific learning leads to song convergence and increased hybridization and introgression.

Since interspecific hybrids in birds usually have lower fitness compared to their parental species due to sterility of heterogametic females [6] or inferior ecological characteristics [28]–[30] (but see, e.g., [18], [31]), the convergence in mating signals resulting in increased hybridization rate is assumed to be maladaptive [6]. From this point of view, it seems interesting that song convergence occurs frequently in many secondary contact zones of closely related birds [8], [12], [23], [32], [33]. This raises the question whether song convergence in sympatry could be adaptive in some respects. One possibility is that song convergence brings an advantage to males while defending their territories against heterospecific males, and thus reduces the costs of interspecific competition [34]. Furthermore, song convergence may be the result of an ecological adaptation to the local acoustic environment [14]–[17] or may arise as a by-product of selection on another trait involved in signal production [35]–[38].

Mechanisms underlying song convergence in secondary contact zones have been studied in detail only in few species including Darwin finches [22], [39], [40], flycatchers *Ficedula* spp. [8], [23] and warblers *Hippolais* spp. [20], [33]. The results of these studies indicate that several of the mechanisms mentioned above may contribute to this phenomenon in different species; however, the evolutionary consequences, effects on the speciation process, and a potential adaptive value of song convergence remain poorly understood.

In this study, we investigated the mechanisms underlying song convergence in the secondary contact zone between two nightingale species, the Thrush Nightingale (*Luscinia luscinia*) and the Common Nightingale (*Luscinia megarhynchos*). These species diverged approximately 1.8 MY ago [41], and during the Holocene got into secondary contact in Central and Eastern Europe, with present distribution areas overlapping in a contact zone spanning from northeastern Germany to the Black Sea [42]. Despite the overall morphological similarity, the species can be distinguished by subtle differences in several wing feather characteristics, plumage coloration, and body size [43], [44]. Both species have similar ecological requirements but partial habitat segregation has been observed in sympatry [45], [46]. The divergence in relative bill size, most likely caused by segregation of feeding niches between the species, has also been documented in areas where both species co-occur [47].

The two nightingale species show strong assortative mating in sympatry. Nonetheless, mixed pairs occasionally arise and produce viable F_1_ hybrids, which can be recognized according to their intermediate morphological characteristics [48]–[50]. Morphological as well as genetic studies suggest that approximately 3–5% of nightingales in sympatry are F_1_ hybrids [47], [48]. The interspecific hybrids have apparently reduced fitness in comparison with their parental species, particularly due to sterility of F1 females according to Haldane’s rule [50], [51]. However, the genetic analyses revealed also a small number of backcross hybrids in sympatric populations, although their precise frequency could not be estimated due to a limited number of genetic markers used [47].

**Figure 1:**
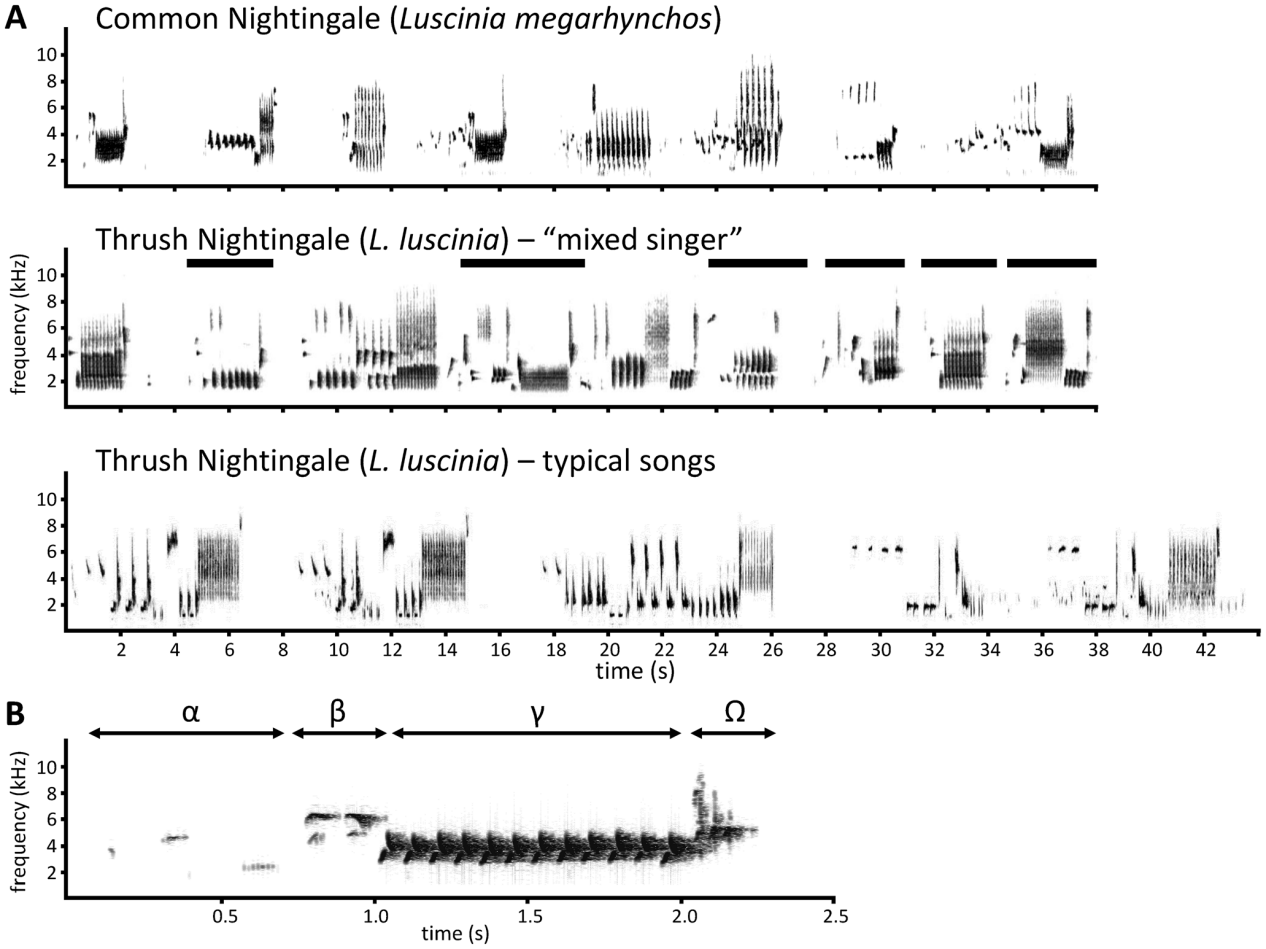
Spectrograms of representative recordings of studied species. Spectrograms of representative recordings of Common Nightingale, Thrush Nightingale “mixed singer”, and allopatric Thrush Nightingale (A), with songs characteristic for Common Nightingale in the mixed singer’s repertoire indicated by horizontal bars. The first Common Nightingale song from A is enlarged (B) to demonstrate the typical song organization of this species, consisting of alpha, beta, gamma and omega parts (after [52]).

The song of both nightingale species belongs to the most complex among songbirds. Nevertheless, there are considerable differences in the song of both species. The repertoire of an average Common Nightingale male consists of some 190 song types [52], [53], the Thrush Nightingale repertoires are substantially smaller (up to 42 song types according to [54], and approx. 40–50 song types per male in our samples; Kipper et al., unpubl. data). The latter species sings songs with a longer duration and a lower song rate [44], [55], and both species differ in typical song organization (Fig. 1A). As in most oscine passerines, songs in nightingales are acquired through social learning from a model (father or neighbour). Since this learning occurs both during juvenile and adult periods of their lives, nightingales are considered ‘open ended learners’ (reviewed in [56], [57]).

Asymmetric song convergence has been described in areas where the two nightingale species co-occur. A relatively large proportion of Thrush Nightingales living in sympatry include song types from the Common Nightingale in their repertoires [45], [48], [55], [58], [59] (Fig. 1A); these birds have been called ‘mixed singers’. On the other hand, singing of heterospecific songs is very rare in the Common Nightingale (only one out of 200 studied males was determined as a mixed singer in [55]).

Here we tested whether introgressive hybridization can account for mixed singing in the sympatric Thrush Nightingales. For this purpose, we performed simultaneous analyses of song, phenotype, and multilocus genetic data from sympatric and allopatric populations of both species, including putative hybrid individuals. We used a semi-automated approach for detecting Common Nightingale song types in recordings of both species. This allowed us for the first time to quantify the proportion of heterospecific songs in repertoires of ‘mixed singers’, as well as to quantitatively compare song composition of interspecific hybrids and pure species.

## Methods

### Ethics Statement

All necessary permits were obtained for the described field studies. The field work and manipulation with birds was approved by the Local Ethic Committee for Scientific Experiments on Animals in Poznan, Poland (permission no. 27/2008) and by the Ministry of Education, Youth and Sport of the Czech Republic (permission no. 9833/2007-30). Only song recordings were obtained from the German Common Nightingale population.

### Field Sampling and Song Recording

The fieldwork was carried out during springs 2007 to 2009 in allopatric and sympatric breeding sites of both species. Allopatric areas were represented by localities in the Czech Republic and Germany for the Common Nightingale and in north-eastern Poland for the Thrush Nightingale. Study sites in sympatry were located in central Poland (see Results and Table S1). All but one sympatric sample were collected in floodplains of Prosna and Warta rivers on the sites with similar densities of both species. One individual with an intermediate phenotype was sampled at the south-western border of sympatry where Common Nightingale dominates. The birds were recorded and captured at the beginning of the breeding season in April and May. They were captured by mist netting, lured into nets (Ecotone, Poland) by playback of commercial recording of either Common Nightingale or Thrush Nightingale [60], depending on the presumed species identity of the respective male. The males were then individually marked by metal and colour rings for later identification. Preliminary species identification of captured birds was based on evaluation of species-specific phenotypic characteristics, including plumage colouration and wing feather measurements [43]. Blood samples for subsequent genetic analyses were collected from each sympatric individual by brachial vein puncture. Samples were stored in pure ethanol until further processing.

The songs were recorded immediately before or within two days after capture (in the same territory) on a digital recorder Marantz PMD660 using a directional Sennheiser ME67 microphone. Altogether, we analysed songs of 41 males including: eleven allopatric Common Nightingales, eight allopatric Thrush Nightingales, nine sympatric Thrush Nightingales, eight sympatric Common Nightingales, and five sympatric males with an intermediate phenotype. Details for individual recordings are given in Table S1. All recordings were obtained during days and evenings (from 6 am to 9 pm), except for six Common Nightingale males from allopatry that were recorded at night.

### Analysis of Songs

Recordings were analysed using the software Avisoft SASLab Pro versions 4.5 to 5 [61]. We analysed approximately 20 minute long recordings of each individual (range 19–23 min; only recordings of two males were shorter, 8 and 14 minutes). The recordings consisted on average of 200 songs per individual in the Common Nightingale (median 204; range 132–265), 148 songs per individual in the Thrush Nightingale (median 148; range 88–206) and 131 songs per individual in males of intermediate phenotype (median 139; range 64–176). Recording length, numbers of analysed songs, results of their assignment to different song type categories (as defined below), and other details are given for each individual in Table S1.

**Figure 2:**
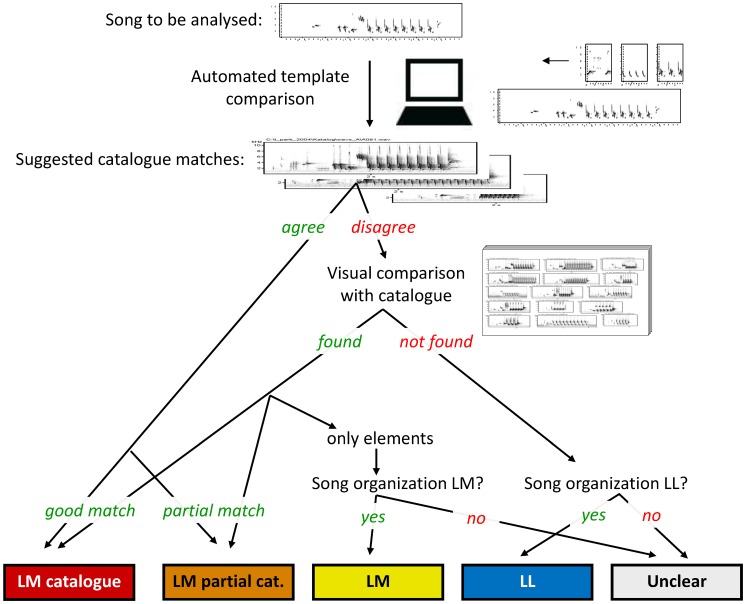
Song analysis workflow: from a recording to category assignment. Visualization of the semi-automated song assignment process comparing each song to be analysed with the catalogue of Common Nightingale song types, and assigning it to one of five song categories (bottom). Visual comparisons were conducted by two people independently of each other. Terms in italics indicate observer decisions (see Methods for details).

To identify ‘mixed singers’ among the recorded males, we compared the singing of each male to a catalogue of songs from Common Nightingales based on several German populations. The catalogue consisted of 425 distinct song types and was derived from analyses of nocturnal singing of 50 Common Nightingales (6 years, 3 populations, 533 successive songs per bird equalling about 1 hr of singing; [62]). Each song in our recordings was compared with the catalogue song types and categorized accordingly (see the workflow summarized in Fig. 2). Nightingales are known for their precise song copying and singing, so that despite their large repertoires, song types can be reliably assigned and compared across individuals, populations, and years [53], [63].

Though the comparison and categorization of songs can be reliably achieved by visual comparisons of spectrograms, we decided to apply a semi-automated procedure to conduct the categorization as objective as possible. For this we used the spectrogram image cross-correlation analysis in Avisoft SASLab Pro and compared each song with templates of song types included in the catalogue (547 templates, each of 0.5 s duration; some song types consisting of two repetitive phrases were represented by two different templates). These templates were selected to be sections of the ‘loudest’ part of the respective song type (i.e., the part with the maximum amplitude), which were likely to match other recordings of the same song type.

To facilitate batch cross-correlations, all recordings were first split into files containing single songs. These files were subsequently converted into a format suitable for the analysis with the following settings: sampling frequency conversion 22.05 kHz (accuracy 256), normalize volume (85%), noise reduction filter (FFT 512, precision 4, threshold −40 db, reduce by 90 db). From each file, a spectrogram file was created (FFT 256, frame size 100, Hamming window, overlap 50%). Matches of the catalogue song templates with each analysed song were evaluated by the function ‘Classify.wav or.son files’ in Avisoft SASLab Pro, and final visual comparisons were facilitated by an Excel macro that allowed a quick inspection of spectrograms of the best-matching song types from the catalogue. Songs that were not identified by this cross-correlation procedure were re-checked by a visual comparison with the catalogue by two scorers independently from each other and without knowledge of the recording origin.

**Figure 3:**
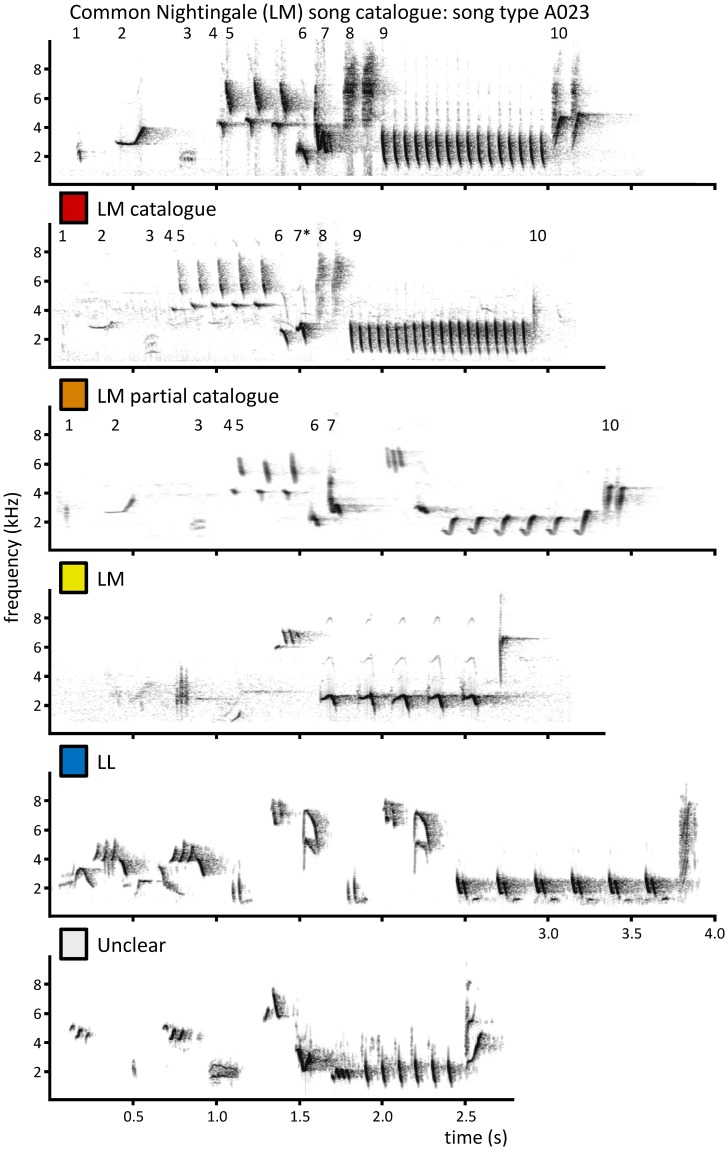
Examples of song categories, taken from repertoires of sympatric Thrush Nightingales. The Common Nightingale song type included in the catalogue (A023), to which the examples for categories “LM catalogue” and “LM partial catalogue” were assigned, is shown on top. Different elements in the catalogue example and their counterparts in Thrush Nightingale songs are numbered; the asterisk indicates similar but distinct variants. Note that a particular song type is characterized by the order of different elements; the number of their repetitions is variable and does not influence the assignment to particular categories. See Methods and Fig. 2 for definitions of song categories and the workflow of category assignment.

Each analysed song was assigned to one of five categories reflecting the degree of similarity to the template song types in the catalogue (see examples in Fig. 3): (1) **‘LM catalogue’** – songs matching a Common Nightingale catalogue song type exactly or resembling it by at least 95% of the element sequence of the catalogue. (2) **‘LM partial catalogue’** – songs similar to a catalogue song type, but differing from it due to absence or difference of some elements, with resemblance of at least 75% of the catalogue song element sequence. (3) **‘LM’** – songs that could not be assigned to a catalogue song type, but did show the typical Common Nightingale song organization (alpha-beta-gamma-omega [52]; see Fig. 1B) and recognizable Common Nightingale catalogue gamma parts. (4) **‘LL’** – songs that could not be assigned to a catalogue song type, showed typical Thrush Nightingale song organization (beginning with a repeated part, no beta-part, no omega) and did not contain any Common Nightingale catalogue gamma parts. (5) **‘Unclear’** – any disputable cases including those of no Common Nightingale song organization, but recognizable gamma parts. Fragmented and poorly recorded song types exceptionally found in our recordings (less than 0.3% of all songs) were excluded from further analyses.

For the final evaluation of song composition of individual birds, each song was assigned to one of the two overall groups, **‘Identified’** and **‘Not identified’**, depending whether it was identified as a song type typical for the Common Nightingale. The ‘Identified’ group included the categories ‘LM catalogue’, ‘LM partial catalogue’ and ‘LM’, whereas the ‘Not identified’ group included categories ‘Unclear’ and ‘LL’. The ‘Identified’ group thus included songs which did not match strictly any song in the catalogue but were most likely of a Common Nightingale origin. The inclusion of this category was thus useful when evaluating the proportion of heterospecific songs sung by Thrush Nightingale mixed singers. Analysing the data with stricter criteria (only ‘LM catalogue’ and ‘LM partial catalogue’ included in the ‘Identified’ group) for evaluation of mixed singing of Thrush Nightingales did not affect the results substantially.

### Genetic Analyses

Genomic DNA from the blood sample was isolated by a DNeasy Tissue Kit (Qiagen) and used for PCR amplification of four autosomal and three Z-linked loci, each containing at least one species-informative single nucleotide polymorphism (SNP) (Table 1). The primers for amplification of Z-linked loci are located in conserved exonic regions of the chicken or zebra finch genome so that they amplify intronic sequences [41], [64], [65]. The autosomal primers amplify transcribed sequences (exons or untranslated regions of mRNA) and were designed according to nightingale transcriptome sequence data (Mořkovský et al., unpublished). Primer sequences and PCR conditions are given in Table S2. One selected species-informative SNP at each locus (except for *ADAMTS6*) was genotyped in all analysed sympatric individuals and 20 control allopatric individuals using ABI PRISM SNaPshot Multiplex Kit (Applied Biosystems). The protocol followed the manufacturer’s instructions, used primers and their concentrations in the reaction are provided in Table S3. Genotypes at the locus *ADAMTS6* were obtained by sequencing the whole PCR product.

**Table 1:** List of seven loci containing species-informative SNPs used for identification of hybrid individuals.

			Species-informative SNPs^2^	
Locus name	Chromosome^1^	Position (Mb)^1^	*L. megarhynchos*	*L. luscinia*
*ADAMTS6*	Z	50.7	T (100%)	C (100%)
*SPINZ-2*	Z	7.5	A (100%)	G (100%)
*TG5287*	Z	64.5	G (100%)	A (100%)
*Lu01*	6	18.5	G (95%), T (5%)	T (97.5%), G (2.5%)
*Lu03*	4	47.1	T (100%)	C (100%)
*Lu04*	1A	55.0	G (100%)	T (100%)
*Lu10*	3	64.8	T (80%), C (20%)	C (95%), T (5%)

1 Position in the zebra finch genome, assembly taeGut3.2.4.

2 The frequency of alleles occurring in each species was determined in a sample of 20 allopatric individuals of both species.

The obtained SNP data were analysed using the program NewHybrids version 1.1b [66] to estimate the posterior probability that individuals in a sample fall into six pre-defined genotype categories: (1) pure Common Nightingale, (2) pure Thrush Nightingale, (3) F_1_ hybrid, (4) F_2_ hybrid, (5) first-generation backcross (BC_1_) hybrid in the direction of Common Nightingale, and (6) BC_1_ hybrid in the direction of Thrush Nightingale. The analysis was performed for allopatric and sympatric individuals pooled, without including prior phenotypic information. The program assumes that the analysed loci are not closely linked to each other. In the absence of physical or genetic map for nightingales, we determined the chromosomal position of each locus in the zebra finch, the only passerine with the known genome (Table 1). All four autosomal loci lie on different chromosomes. The three Z-linked loci are located in different regions of the Z chromosome at least 13.8 Mb apart, a distance roughly corresponding to 20 cM assuming that the recombination rate on the zebra finch Z chromosome is 1.43 cM/Mb [67]. As the studied loci are not closely linked in the zebra finch genome, we can reason that they are unlikely to be all linked in the nightingale genome.

To identify parental species of F_1_ hybrids, we sequenced a 525 bp fragment of the maternally inherited mitochondrial gene for NADH dehydrogenase subunit 2 (ND2) using primers and PCR conditions published in [50]. The high quality 193 bp sequence obtained from all hybrid individuals was then compared to previously obtained homologous sequences from 15 individuals of the Common Nightingale and 17 individuals of the Thrush Nightingale [50]. The analysed sequences are sufficiently divergent between the nightingale species, differing by 10 fixed single nucleotide polymorphisms (SNPs), to serve for unambiguous identification of the maternal species.

### Statistical Analysis

All statistical analyses were performed in R 2.15.0 [68]. For comparison of song rate between species, we used a non-parametric test (Mann-Whitney U test). To test if there was any difference in song composition between allopatric and sympatric individuals of the same species, we used generalized linear models (GLM, quasibinomial family due to overdispersion, logit link function). Proportions of identified/not identified songs were used as a response variable, and the area of occurrence (sympatry or allopatry) was used as a categorical explanatory variable. The same statistical approach was used for the evaluation of differences in song composition between the sympatric Thrush Nightingale males and F_1_ hybrids and Common Nightingale males, respectively.

## Results

### Analyses of Songs in Sympatric and Allopatric Populations

Altogether, we analysed 6984 songs from 41 males. Of these, 3798 songs were from 19 Common Nightingale males, 2531 songs from 17 Thrush Nightingale males, and 655 songs from 5 males with intermediate phenotype. Thrush Nightingale males sung with a significantly lower rate than Common Nightingale males: on average 7.2 (range 4.2–10.3) songs per minute vs. 9.8 (range 6.1–14.9) songs per minute; Mann–Whitney U test; N_1_ = 17, N_2_ = 19, U = 53, exact p = 0.00034.

**Figure 4:**
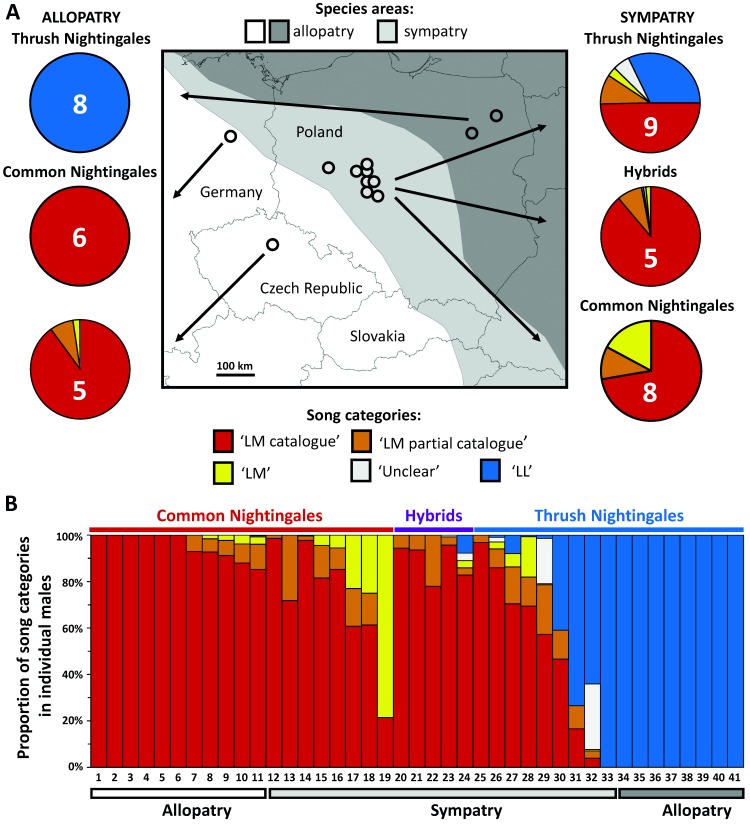
Proportions of song categories in Nightingale recordings from sympatry and allopatry. Proportions of song categories in song samples of allopatric and sympatric Common and Thrush Nightingales. Results averaged for different taxa (pure species and hybrids) and origin (allopatry vs. sympatry) are shown together with a map showing areas of sympatry and allopatry in the studied region (after [47]) and geographic origin of studied males (A). Numbers of analysed males are given in each pie chart; data for allopatric Common Nightingales are shown separately for the German population (the source for the song type catalogue), and for the Czech population. Repertoire compositions of individual analysed birds, ordered according to source region, taxon, and proportions of Common Nightingale song types, are shown in stack bars (B).

One of the Common Nightingale populations from allopatry (Germany) belonged to the populations used for deriving the catalogue. Accordingly, their songs corresponded completely to catalogue song types (Fig. 4A). The other population of males from Common Nightingale allopatry matched the catalogue song types similarly well: on average 98% (range 96–100%; Fig. 4 A,B) of more than 1100 songs of allopatric Common Nightingale males from the Czech Republic were also identified in the catalogue (i.e. category ‘LM catalogue’ or category ‘LM partial catalogue’). Of the remaining 27 songs, 93% were categorized as species-typical Common Nightingale song (‘LM’), and only two songs of one male were categorized as either ‘Unclear’ or ‘LL’. Common Nightingale males in sympatry sung on average 83% (range 21–100%; Fig. 4 A, B) of songs identifiable in the catalogue (category ‘LM catalogue’, ‘LM partial catalogue’). All remaining songs were categorized as ‘LM’.

There were no ‘mixed singers’ among the analysed Thrush Nightingale males from allopatry, all their songs were categorized as the song types typical for that species (category ‘LL’). In contrast, only one out of nine analysed Thrush Nightingales from the sympatric region seemed not to use any Common Nightingale song types and had apparently a pure Thrush Nightingale repertoire. Seven out of nine males were ‘mixed singers’, i.e., they used some Common Nightingale song types found in the catalogue and often also additional song types with characteristics typical for the Common Nightingale, together with typical Thrush Nightingale songs in their repertoires. The proportion of these heterospecific songs varied substantially among individuals (on average 62%, range 7–94%), and song categories were represented differently in different individuals (Fig. 4B). Interestingly, the repertoire of the remaining one Thrush Nightingale male contained exclusively Common Nightingale song types. The differences in the proportion of identified heterospecific song types between sympatric and allopatric Thrush Nightingale males were highly significant (GLM, DF1 = 1, DF2 = 15, F = 25.94, p = 0.00013), as was the difference in the proportion of Common Nightingale song types in repertoires of sympatric Thrush Nightingale and Common Nightingale males (GLM, DF1 = 1, DF2 = 15, F = 14.56, p = 0.0016). In the latter species, no heterospecific song types were recorded in sympatry. Thus, sympatric and allopatric males did not differ significantly in the proportion of Common Nightingale song types (GLM, DF1 = 1, DF2 = 17, F = 2.69, p = 0.12).

Two out of five intermediate-phenotype birds were evaluated as ‘mixed singers’, repertoires of which were dominated by Common Nightingale song types: 86 and 99% respectively (Fig. 4), i.e., higher than in most Thrush Nightingale mixed singers. Three intermediate-phenotype birds apparently used only Common Nightingale song types in their repertoires. Thus, there was a clear trend that intermediate-phenotype males use higher proportion of identified Common Nightingale song types than sympatric Thrush Nightingale males, although marginally non-significant (GLM, DF1 = 1, DF2 = 12, F = 4.63, p = 0.052).

### Taxon Assignment of Sympatric Nightingales

Using seven species-informative SNP markers we genotyped 20 individuals of each nightingale species from allopatric regions, which should represent pure species, and 22 sympatric individuals with analysed song, including eight with Common Nightingale phenotypic traits, nine of Thrush Nightingale and five of intermediate phenotype. Analysis of the obtained SNP data in NewHybrids confirmed that all allopatric individuals represented pure species (posterior probability >99%). The eight Common Nightingales as well as nine Thrush Nightingales from sympatry were also classified as pure parental species (posterior probability >99%). All five individuals with intermediate phenotype were classified as F_1_ hybrids (posterior probability >95%). Analysis in NewHybrids suggested that the studied Thrush Nightingale ‘mixed singers’ were not BC_1_ hybrids. Furthermore, as five of the seven analysed SNPs were species-specific, and none of the ‘mixed singers’ was heterozygous at any of these loci, it is unlikely that these males were BC_2_ or BC_3_ hybrids, which are expected to show on average 25% and 12.5% heterozygous loci, respectively.

To determine the species identity of parents of F_1_ hybrids, we compared the partial *ND2* sequences from all five analysed hybrid individuals with reference individuals of both parental species. Sequences from all F_1_ hybrids randomly chosen in the sympatric population were of Thrush Nightingale origin according to ten species-specific SNPs occurring in this sequence. Accordingly, all F_1_ hybrids originated from mating of a Thrush Nightingale female with a Common Nightingale male.

## Discussion

Our study brings further evidence that European nightingale species are an excellent model for studying ecological, evolutionary and behavioural consequences of secondary contact and hybridization of closely related bird species. By combining genetics, morphology, and song analyses, we gained a better insight into causes and consequences of the phenomenon of song convergence in their contact zone.

With our semi-automated assigning approach we were able to quantify the occurrence of mixed singers among the Thrush Nightingale males in the area of sympatry with the Common Nightingale. In congruence with former studies [45], [48], [55], we showed that Thrush Nightingale mixed singers are common in sympatric populations. Frequencies of mixed singers among sympatric Thrush Nightingale males estimated in previous studies were 28% [55], 44% [45], and 56% [48]. In our dataset, this frequency was even higher: seven out of nine sympatric Thrush Nightingale males analysed in this study were mixed singers, one sang only Common Nightingale songs, and only one had a pure Thrush Nightingale repertoire.

The variation in estimates of mixed singer proportions in sympatric populations could be caused not only by differences among studied populations, but also by a higher sensitivity of our method to detect mixed singers. Using this new approach, we quantified the proportion of Common Nightingale song types in the repertoire of individual Thrush Nightingale mixed singers and hybrids for the first time, and showed that it varied considerably among individual birds. Particularly high proportions of Common Nightingale song types were observed in repertoires of the five genetically confirmed F_1_ hybrid males: three sung purely Common Nightingale songs, while two used rarely some Thrush Nightingale song types. This might be related to the fact that all hybrid males were descendants of Common Nightingale fathers, assuming that nightingale juveniles learn songs predominantly from their fathers.

In principle, introgressive hybridization could play an important role in song convergence if the tendency for heterospecific song learning is heritable. The results of our genetic analysis, however, do not support this hypothesis. The Thrush Nightingale mixed singers in our study were not early-generation backcross hybrids and although we cannot rule out that they possess at least some introgressed loci, we can argue that such old introgression events are unlikely to explain the asymmetrical song convergence in sympatry. Although genetic introgression between the species occurs in both directions (as recently demonstrated in [41]), mixed singers were observed exclusively among sympatric Thrush Nightingales.

Two other suggested ecological mechanisms promoting song convergence, namely (1) adaptations to the local acoustic environment [5], [14], [16], and (2) adaptive changes in morphological traits affecting song production [35], [37], [38], are also unlikely to explain the frequent occurrence of mixed singers in the Thrush Nightingale. Such mechanisms are expected to result in quantitative changes in temporal or frequency parameters of the song [14], [16], [20], [69]. They are, however, unlikely to cause acquisition of completely different song types, as happens in nightingales.

Interspecific cultural transmission caused by heterospecific learning, shown to cause mixed singing in *Ficedula* flycatchers [8] or Darwin finches [22], [39], [40], thus remains the most likely explanation for the frequent occurrence of mixed singers in the sympatric populations of the Thrush Nightingale. The question still remains whether heterospecific learning is adaptive or not, and what causes the asymmetry of heterospecific song copying observed our study species. Song learning experiments in captivity [51] have shown that both species are able to learn the heterospecific song when reared in isolation. Despite that, mixed singing in nature is common in the Thrush Nightingale, but almost absent in the Common Nightingale.

The Thrush Nightingale seems more plastic in song learning from their territorial neighbours. This is supported by higher repertoire similarity among neighbouring males when compared to distant males [54], [70]. Such pattern has not been observed for the Common Nightingale, where song repertoires are relatively similar even between distant populations [52], [53], [70]. Different plasticity in song learning from neighbours could thus partly explain the higher incidence of mixed singing in the Thrush Nightingale. Another possible mechanism likely resulting in strong and asymmetrical cultural transmission includes social pairing of Thrush Nightingale females and Common Nightingale males, with extra-pair offspring sired by Thrush Nightingale males (as observed in flycatchers [71]). Juveniles from such clutches would be genetically pure Thrush Nightingales but learn heterospecific songs. Unfortunately, no data on extra-pair paternity or frequency of mixed pairs are available from the nightingale contact zone.

It has been suggested that song convergence in sympatry may facilitate the development of interspecific territoriality and thus decrease the intensity of interspecific competition [34], [72]. Such convergence is likely to be asymmetric; advantageous in the dominant species and maladaptive in the subordinate species [73]. Thrush Nightingales are considered dominant in interspecific competition with Common Nightingales [47], it is thus possible that mixed singers are favoured thanks to more efficient territory defence against heterospecific males. This seems supported by anecdotal observations of males switching from apparently pure Thrush Nightingale to mixed song when counter-singing with a Common Nightingale territorial neighbour [48].

Similar to the song learning in males, female preferences for song also result from a combination of genetic and cultural evolution [74]. Therefore, juvenile females in areas of sympatry may acquire future song preferences by imprinting from both conspecific and heterospecific males. For such females, the more variable song of mixed singers may function as an additive attractant enriching an otherwise species-specific song display. The five hybrids analysed in our study came from crosses between female Thrush Nightingales and male Common Nightingales. Assuming that mate choice in nightingales is strongly based on song characteristics, this indicates that at least some female Thrush Nightingales may have a general preference for more complex Common Nightingale song (similarly as demonstrated for male ornamentation in bright-coloured estrildid finch species [75]). If mixed songs in Thrush Nightingales are more attractive for conspecific females, the proportion of mixed singers among males may increase in time and preferences for mixed singing can eventually establish in a particular population [76]. The high proportion of mixed singers among Thrush Nightingale males in sympatry might be a result of such process.

In contrast to previous studies in *Ficedula* flycatchers [8], [23], our results suggest that song convergence in the Thrush Nightingale does not substantially increase the hybridization rate with the Common Nightingale. If mixed singing significantly increased the probability of interspecific hybridization, we would expect elevated hybridization rate between Common Nightingale females and Thrush Nightingale males. Although genetic analyses confirm that hybridization in this direction is possible (Reifová, unpubl. data.), and breeding experiments in captivity proved that F_1_ hybrids in both directions are fully viable [51], all hybrids analysed in our study came from the opposite cross. Even if the mixed singers hybridize with heterospecific females slightly more often than males with pure Thrush Nightingale song, this frequency is likely to be very low and does not exceed the frequency of interspecific hybridization in the opposite direction. This could explain why mixed singers are more common in the nightingale then in flycatcher hybrid zone [8].

It is apparent that causes as well as evolutionary consequences of convergence in acoustic signalling substantially vary in different model systems [77]–[79]. Hybridization and introgression or adaptation to habitat characteristics may play key roles in signal convergence in various vertebrate taxa apart from songbirds, including frogs [80] or non-passerine and suboscine birds [16], [19]. Acquisition of signals by learning frequently leads to convergence at intraspecific level, as documented in various groups of mammals and birds [81]. However, vocal convergence of different species resulting from heterospecific learning seems to be a phenomenon particularly important, and best documented, for songbirds. The results of our study suggest that in Thrush Nightingales, mixed singing is not the result of hybridization and introgression. Instead, it may have adaptive value; the fitness loss of mixed singers due to interspecific hybridization is apparently lower than fitness gain due to improved territorial defence and/or increased attractiveness for conspecific females. Further experimental work including playback experiments and testing of female preferences in the nightingale hybrid zone may improve our understanding of selection forces responsible for asymmetrical song convergence in these species.

## Supporting Information

Table S1 **List of all nightingale males included in song analyses, with information about recordings (date, duration and localities), taxon, number of songs per minute and number of songs in each category per each individual.**
(DOC)

Table S2 **Primers and PCR conditions for amplification of analysed loci.**
(DOC)

Table S3 **SNaPshot primers and their concentrations in the reaction.**
(DOC)
