# Supplementary material for: The Causes and Evolutionary Consequences of Mixed Singing in Two Hybridizing Songbird Species (Luscinia spp.)
Source: PLoS One. 2013 Apr 5;8(4):e60172. doi: 10.1371/journal.pone.0060172 (PMC3618175; doi:10.1371/journal.pone.0060172)
Supplement: Table S1 — List of all nightingale males included in song analyses, with information about recordings (date, duration and localities), taxon, number of songs per minute and number of songs in each category per each individual. (DOC) [file pone.0060172.s001.doc]

**Supplementary Table 1.** List of all nightingale males included in song analyses, with information about recordings (date, duration and localities), taxon, number of songs per minute and number of songs in each category per each individual.

|  |  |  |  |  | Coordinates | |  |  | Categories | | | | | |  |
| --- | --- | --- | --- | --- | --- | --- | --- | --- | --- | --- | --- | --- | --- | --- | --- |
| Number | Taxon | Duration | Date | Locality | N | E | No songs | Song  rate | LM catalogue | LM partial catalogue | LM | LL | Unclear | Excluded | % LM songs |
| **Allopatry: Common Nightingales** | | | |  |  |  |  |  |  |  |  |  |  |  |  |
| 1 | LM | 21:30 | April 2008 | Berlin (D) | 52°29.5' | 13°28.3' | 150 | 6.9 | 150 | 0 | 0 | 0 | 0 | 0 | 100 |
| 2 | LM | 20:00 | April 2008 | Berlin (D) | 52°29.0' | 13°29.0' | 143 | 7.2 | 143 | 0 | 0 | 0 | 0 | 0 | 100 |
| 3 | LM | 20:00 | April 2008 | Berlin (D) | 52°29.1' | 13°28.2' | 213 | 10.7 | 213 | 0 | 0 | 0 | 0 | 0 | 100 |
| 4 | LM | 20:00 | May 2008 | Berlin (D) | 52°29.4' | 13°27.7' | 217 | 10.9 | 217 | 0 | 0 | 0 | 0 | 0 | 100 |
| 5 | LM | 21:30 | May 2009 | Berlin (D) | 52°29.4' | 13°27.7' | 132 | 6.1 | 132 | 0 | 0 | 0 | 0 | 0 | 100 |
| 6 | LM | 20:00 | May 2009 | Berlin (D) | 52°29.4' | 13°28.3' | 144 | 7.2 | 144 | 0 | 0 | 0 | 0 | 0 | 100 |
| 7 | LM | 20:10 | May 2009 | Předměřice n Jiz. (CZ) | 50°14.5' | 14°45.4' | 199 | 9.9 | 183 | 14 | 0 | 0 | 0 | 2 | 100 |
| 8 | LM | 20:35 | May 2009 | Předměřice n Jiz. (CZ) | 50°14.6' | 14°46.1' | 190 | 9.2 | 176 | 11 | 3 | 0 | 0 | 0 | 100 |
| 9 | LM | 23:00 | May 2009 | Předměřice n Jiz. (CZ) | 50°14.9' | 14°46.5' | 261 | 11.3 | 237 | 17 | 6 | 0 | 0 | 1 | 100 |
| 10 | LM | 21:00 | May 2009 | Předměřice n Jiz. (CZ) | 50°15.5' | 14°45.9' | 212 | 10.1 | 184 | 17 | 8 | 0 | 0 | 3 | 100 |
| 11 | LM | 23:00 | May 2009 | Předměřice n Jiz. (CZ) | 50°15.9' | 14°46.0' | 251 | 10.9 | 214 | 27 | 8 | 1 | 1 | 0 | 99 |
| **Sympatry: Common Nightingales** | | | |  |  |  |  |  |  |  |  |  |  |  |  |
| 12 | LM | 20:00 | May 2008 | Czolnochów (PL) | 52°03.0' | 17°42.7' | 204 | 10.2 | 200 | 3 | 0 | 0 | 0 | 1 | 100 |
| 13 | LM | 20:00 | May 2008 | Czolnochów (PL) | 52°02.6' | 17°43.5' | 161 | 8.1 | 115 | 45 | 0 | 0 | 0 | 1 | 100 |
| 14 | LM | 20:21 | May 2008 | Chocz (PL) | 51°58.7' | 17°51.1' | 259 | 12.7 | 252 | 5 | 1 | 0 | 0 | 1 | 100 |
| 15 | LM | 23:00 | May 2008 | Czolnochów (PL) | 52°02.5' | 17°43.8' | 181 | 7.9 | 146 | 25 | 8 | 0 | 0 | 2 | 100 |
| 16 | LM | 14:34 | May 2008 | Olobok(PL) | 51°38.4' | 18°04.4' | 217 | 14.9 | 185 | 20 | 12 | 0 | 0 | 0 | 100 |
| 17 | LM | 20:25 | May 2008 | Chocz (PL) | 51°58.7' | 17°51.2' | 265 | 13.0 | 161 | 43 | 61 | 0 | 0 | 0 | 100 |
| 18 | LM | 20:15 | May 2008 | Wola Droszewska(PL) | 51°37.9' | 18°04.8' | 212 | 10.5 | 130 | 29 | 53 | 0 | 0 | 0 | 100 |
| 19 | LM | 20:00 | May 2008 | Pogorzelica (PL) | 52°08.5' | 17°36.1' | 187 | 9.4 | 40 | 0 | 147 | 0 | 0 | 0 | 100 |
| **Sympatry: Interspecific hybrids** | | | |  |  |  |  |  |  |  |  |  |  |  |  |
| 20 | HYB | 20:00 | May 2007 | Koscian (PL) | 52°08.3' | 16°33.6' | 176 | 8.8 | 166 | 10 | 0 | 0 | 0 | 0 | 100 |
| 21 | HYB | 21:25 | May 2008 | Pogorzelica (PL) | 52°08.3' | 17°35.5' | 140 | 6.5 | 131 | 9 | 0 | 0 | 0 | 0 | 100 |
| 22 | HYB | 20:00 | May 2008 | Ruda Komorska (PL) | 52°07.1' | 17°39.9' | 136 | 6.8 | 106 | 30 | 0 | 0 | 0 | 0 | 100 |
| 23 | HYB | 21:00 | May 2008 | Raduchów (PL) | 51°34.8' | 18°09.8' | 139 | 6.6 | 133 | 5 | 0 | 0 | 1 | 0 | 99 |
| 24 | HYB | 8:11 | May 2009 | Prusinów (PL) | 52°02.9' | 17°42.2' | 64 | 7.8 | 53 | 2 | 2 | 5 | 2 | 0 | 89 |

| Supplementary Table 1 (continued) | | | |  | Coordinates | |  |  | Categories | | | | | |  |
| --- | --- | --- | --- | --- | --- | --- | --- | --- | --- | --- | --- | --- | --- | --- | --- |
| Number | Taxon | Duration | Date | Locality | N | E | No songs | Song  rate | LM catalogue | LM partial catalogue | LM | LL | Unclear | Excluded | % LM songs |
| **Sympatry: Thrush Nightingales** | | | |  |  |  |  |  |  |  |  |  |  |  |  |
| 25 | LL | 20:10 | May 2008 | Przystajnia (PL) | 51°35.1' | 18°10.4' | 158 | 7.8 | 151 | 5 | 0 | 0 | 0 | 2 | 100 |
| 26 | LL | 22:00 | May 2009 | Czolnochów (PL) | 52°02.5' | 17°43.6' | 200 | 9.1 | 172 | 16 | 6 | 2 | 4 | 0 | 97 |
| 27 | LL | 19:36 | May 2008 | Prusinów (PL) | 52°03.0' | 17°42.6' | 177 | 9.0 | 124 | 28 | 10 | 14 | 0 | 1 | 92 |
| 28 | LL | 21:00 | May 2009 | Chocz site 1 (PL) | 51°58.1' | 17°52.3' | 148 | 7.0 | 100 | 18 | 25 | 1 | 0 | 4 | 99 |
| 29 | LL | 20:00 | May 2008 | Kwileň (PL) | 51°59.0' | 17°50.6' | 206 | 10.3 | 118 | 44 | 1 | 3 | 40 | 0 | 79 |
| 30 | LL | 21:00 | May 2009 | Chocz site 2 (PL) | 51°58.7' | 17°51.2' | 88 | 4.2 | 41 | 11 | 0 | 36 | 0 | 0 | 59 |
| 31 | LL | 20:05 | May 2008 | Chocz (PL) | 51°58.3' | 17°51.9' | 151 | 7.5 | 25 | 15 | 0 | 111 | 0 | 0 | 26 |
| 32 | LL | 20:05 | May 2008 | Pogorzelica (PL) | 52°08.5' | 17°35.9' | 157 | 7.8 | 6 | 5 | 1 | 100 | 44 | 1 | 8 |
| 33 | LL | 20:00 | May 2008 | Czolnochów (PL) | 52°02.5' | 17°43.8' | 119 | 6.0 | 0 | 0 | 0 | 119 | 0 | 0 | 0 |
| **Allopatry: Thrush Nightingales** | | | |  |  |  |  |  |  |  |  |  |  |  |  |
| 34 | LL | 20:01 | May 2009 | Wizna (PL) | 53°11.7' | 22°22.9' | 116 | 5.8 | 0 | 0 | 0 | 116 | 0 | 0 | 0 |
| 35 | LL | 20:00 | May 2009 | Wizna (PL) | 53°11.8' | 22°22.1' | 112 | 5.6 | 0 | 0 | 0 | 112 | 0 | 0 | 0 |
| 36 | LL | 19:45 | May 2009 | Wizna (PL) | 53°11.9' | 22°22.1' | 118 | 6.0 | 0 | 0 | 0 | 118 | 0 | 0 | 0 |
| 37 | LL | 20:05 | May 2009 | Goniadz (PL) | 53°34.2' | 22°44.8' | 180 | 9.0 | 0 | 0 | 0 | 180 | 0 | 0 | 0 |
| 38 | LL | 19:59 | May 2009 | Goniadz (PL) | 53°34.3' | 22°44.9' | 146 | 7.3 | 0 | 0 | 0 | 146 | 0 | 0 | 0 |
| 39 | LL | 23:28 | May 2009 | Wizna (PL) | 53°11.7' | 22°23.0' | 188 | 8.0 | 0 | 0 | 0 | 188 | 0 | 0 | 0 |
| 40 | LL | 20:00 | May 2009 | Wizna (PL) | 53°11.8' | 22°22.5' | 132 | 6.6 | 0 | 0 | 0 | 132 | 0 | 0 | 0 |
| 41 | LL | 22:13 | May 2009 | Wizna (PL) | 53°12.0' | 22°22.1' | 135 | 6.1 | 0 | 0 | 0 | 135 | 0 | 0 | 0 |
