# Supplementary material for: The Causes and Evolutionary Consequences of Mixed Singing in Two Hybridizing Songbird Species (Luscinia spp.)
Source: PLoS One. 2013 Apr 5;8(4):e60172. doi: 10.1371/journal.pone.0060172 (PMC3618175; doi:10.1371/journal.pone.0060172)
Supplement: Table S2 — Primers and PCR conditions for amplification of analysed loci. (DOC) [file pone.0060172.s002.doc]

**Supplementary Table 2.** Primers and PCR conditions for amplification of analysed loci.

|  | **Product** | **Primer sequences (5’-3’)** | | |  | |
| --- | --- | --- | --- | --- | --- | --- |
| **Locus name** | **size (bp)** | **Forward primer** | **Reverse primer** | | **References** | |
| *ADAMTS6* | 700 | GGAGAGAATGGATTTCTGCC | | TGATTCCAGTCTAGGAAACG | [65] |  |
| *SPINZ-2* | 1000 | ATCATTGTGTCTGCCAGGTG | | CCTCAAGACTTCCCCAAAGA | [41, 64] |  |
| *TG5287* | 900 | GAGTCTTTATCGAGAGCACTGTGA | | AATAGCGCCAGAAAGTTTGC | Reifová et al. *in prep.* |  |
| *Lu01* | 312 | GCACATTTCATGGTCTCCAA | | TGAAAAACAGGCTGCAGAAA | This study |  |
| *Lu03* | 568 | TTCTTGGCATAAATCCATCTGA | | ATGCAGTTGCTTCTGCAGTG | This study |  |
| *Lu04* | 205 | CCATGTCAGATTTAGCATCCA | | AGTGGTTTTCAGAGCACAGG | This study |  |
| *Lu10* | 272 | CAACCAAATGAAAGCTACTGGA | | CTCATGCAGACACAACTGTGAA | This study |  |

All PCR reactions of 25 µl volume consisted of MgCl_2-_free buffer (1x, Promega), MgCl_2_ (2 mM), primers (0.33 µM), dNTPs (0.2 mM), Taq Polymerase (0.625 unit per reaction), and DNA template (ca 2 ng per reaction). The PCR cycle, identical for all loci, consisted of an initial denaturation step of 2 min at 95 °C, followed by six touchdown cycles with denaturation at 94 °C for 30 s, annealing for 30 s at temperatures gradually decreasing from 60 to 55 °C (by 1 °C per cycle) and amplification step at 72 °C for 60 s, followed by 33 cycles of identical timing with annealing temperature of 55 °C, and a final amplification at 72 °C for 5 min.
