# Supplementary material for: The Causes and Evolutionary Consequences of Mixed Singing in Two Hybridizing Songbird Species (Luscinia spp.)
Source: PLoS One. 2013 Apr 5;8(4):e60172. doi: 10.1371/journal.pone.0060172 (PMC3618175; doi:10.1371/journal.pone.0060172)
Supplement: Table S3 — SNaPshot primers and their concentrations in the reaction. (DOC) [file pone.0060172.s003.doc]

**Supplementary Table 3.** SNaPshot primers and their concentrations in the reaction.

| **Locus name** | **SNaPshot primer (5'-3')** | **Length** | **Orientation** | **Concentration in reaction (nM)** |
| --- | --- | --- | --- | --- |
| *TG5287* | TGTCAGGTTTAATTTTTTATTTGCAGTAGC | 30 bp | F | 110 |
| *SPINZ-2* | AGGGAGTACAAGAATTCATTGTTGTTTTAACAACATTCTG | 40 bp | F | 55.5 |
| *Lu01* | (GACT)_7_CTCGGTCACTGAGTTCTTTCAG | 50 bp | F | 110 |
| *Lu03* | (GACT)_8_AATTACACTGATTATACATTCACC | 56 bp | F | 222 |
| *Lu04* | (GACT)_9_ATCACTACATCAGAGCCTCCTGGGAA | 62 bp | F | 55.5 |
| *Lu10* | (GACT)_10_TCCCTTTCTTAATAGGAAATTACTACCT | 68 bp | F | 55.5 |

The protocol (including 1. preparation of sample reactions, including a reaction with control DNA and control primer; 2. performing SNaPshot reactions by thermal cycling and post-extension treatment of products; 3. electrophoresing the products on an ABi 3130 Genetic Analyser) followed manufacturer’s instructions for the ABI PRISM SNaPshot Multiplex Kit (Applied Biosystems).
